# Re-examination of old truths: replication of a study to measure the incidence of lactational mastitis in breastfeeding women

**DOI:** 10.1186/1746-4358-8-2

**Published:** 2013-04-20

**Authors:** Linda J Kvist

**Affiliations:** 1 Department of Health Sciences, Lund University, Sweden, Helsingborg Hospital, Helsingborg, Sweden

**Keywords:** Lactational mastitis, Incidence, Causes, Antibiotics

## Abstract

**Background:**

The reported incidence of lactational mastitis varies greatly; the single highest reported incidence in the scientific literature is 33%. The purpose of this study was to collect data regarding incidence and experiences of lactational mastitis from women attending a meeting of lactation specialists and to compare findings in a similar population reported in 1990 by Riordan and Nichols.

**Methods:**

A retrospective questionnaire study was carried out with a group of Danish lactation specialists in 2011. The questionnaire was constructed to replicate that used in 1990 and included questions about occurrence of mastitis, the infant’s age, breast segments afflicted, examination by a physician, use of antibiotics and possible causes of the illness.

**Results:**

As in the earlier research, respondents in this study reported a 33% occurrence of lactational mastitis. This cannot however, be considered as the incidence of mastitis. In order to state the incidence it is necessary to impose a time limit for the collection of data and to know the size of the population at risk. Incomplete emptying of the breast was the factor most frequently cited as the cause of mastitis.

**Conclusions:**

Researchers must strive to be as exact as possible when reporting definitions and incidences of mastitis and should attempt to identify the true population at risk – in this case, all women who were breastfeeding in the uptake area under study, during a specified time limit. Well-designed studies in different global locations are needed before any conclusions can been drawn about the range of incidences of mastitis.

## Background

In order to build knowledge and theories, the scientific method requires that we either integrate earlier findings with new or alternatively that we challenge previous knowledge if we find that new findings contradict the old. There is a tacit understanding in science that “once is not enough” which implies that experiments should be replicated in order to verify findings. Popper wrote that we do not take our observations seriously or treat them as scientific until we have reproduced and tested them [1]. The reason why description of the methods used in research papers is so important is that it should be possible for other researchers to set up a similar experiment to check the viability of previous results. Despite this, it is rare that replication studies are carried out.

As long ago as 1995, researchers drew attention to the fact that studies regarding incidence and prevalence of mastitis were lacking, in part due to the problem of collecting trustworthy data [2]. These problems remained unsolved ten years later [3]. Despite this, the incidence of lactational mastitis is cited in almost all studies published on the subject.

### Incidence

Incidence reports the number of new cases of a disease in a defined population during a specific time [4]. The most commonly used time period is one year but this is not a strict rule. The incidence rate is the number of *new cases* divided by the number in the *population at risk*. Therefore, in order to report the incidence rate of mastitis in breastfeeding women, it is first necessary to know the size of the population at risk, that is the population of breastfeeding women. Although most countries can give a reasonable estimation of the percentage of women who breastfeed their babies, numbers of women currently breastfeeding is a statistic that is not readily available since the population under study is in constant change by virtue of the fact that as some mothers cease breastfeeding their babies others have just given birth and are initiating breastfeeding.

### Incidence rates in the literature

Reported figures vary greatly between countries and continents and may also differ over time. It is not uncommon for reports of mastitis incidence to be based on a breastfeeding cohort, which is assumed to be the population at risk. This can be problematical since a study cohort may have been selected for a specific research purpose and may not necessarily be a representative sample of all breastfeeding women in the study’s uptake area.

Many Australian studies have considered the incidence rate of mastitis; in 1995 researchers reported an incidence rate of 4.9%, which was based on an estimation of the number of women likely to be breastfeeding at three months postpartum [2]. Another Australian research group based their estimation of a 20% incidence on a cohort of 1075 postpartum women, but the number “at risk” was not considered [5]. A more recent Australian study of 1193 women estimated an incidence density using the number of completed weeks of women breastfeeding in four-week blocks and found a cumulative incidence in the first six breastfeeding months of 17.3% [6]. In the USA researchers followed a cohort of 840 women to 12 weeks postpartum and calculated that within the cohort, the overall incidence rate was 8.1% [7]. New Zealand researchers followed a cohort of 350 women up to one year postpartum and an incidence rate of 23.7% was reported [8]. It is unclear whether the population at risk was all mothers who had continued to breastfeed to one year postpartum. A cumulative incidence as high as 27% has also been reported from Australia [9].

In Europe, a Swedish study showed a mastitis incidence of 6%, which was based on breastfeeding statistics that allowed an estimate of the population at risk, which was considered as all breastfeeding women in the study’s up-take area during the time of data collection [10].

An early American study by Riordan and Nichols reported an incidence of 33% in 1990, in a descriptive study of lactational mastitis in long-term breastfeeding women and this is the single highest incidence reported in the scientific literature [11]. The population in Riordan and Nichols study comprised of three groups of participants at conferences sponsored by the International Lactation Consultant Association (ILCA) and La Leche League International (LLLI). Women were eligible for participation if they had ever breastfed. One of the questions that the study aimed to answer was “*What is the incidence of mastitis in long-term breastfeeding women”?*

The population at risk was seen in Riordan and Nichols’ study as all the women who were attending the conferences and who had ever breastfed. The cases of mastitis that were analysed were cases that the study population had experienced during breastfeeding of their children. For some of the analyses in their study, the authors included only those reporting breastfeeding during the previous five years. However, for the analysis of incidence, the whole study population was used. This is a statistic that is difficult to extract from the text but since the authors wrote that one-third of the women reported having mastitis with their last breastfed child and this number was 60, it appears that the population on which the calculation of incidence was based must have been 180.

Since the incidence of mastitis in the Riordan and Nichols’ study is the highest incidence ever reported, and is commonly cited [5,7,8,10], it was of interest to attempt to replicate their investigation and to compare findings.

The aim of this study was to collect data regarding incidence and experiences of lactational mastitis from women attending a meeting of lactation specialists and to compare findings in a similar population to that reported in 1990 by Riordan and Nichols.

## Method

This was a retrospective questionnaire study carried out at a meeting of lactation specialists in Denmark, 2011. Mastitis was defined as any combination of breast tension, erythema, pain and fever in association with breastfeeding.

### Questionnaire and sample

A questionnaire was constructed to answer most of the questions posed in the original research by Riordan and Nichols [11] and consisted of ten questions. The respondents (*n* = 90) were attending a meeting in October 2011 which was arranged by the Danish Association of Certified Lactation Consultants (DACLC). The majority of the respondents were certified lactation consultants and all were interested and active in giving advice and support to women during their breastfeeding period. The author gave verbal information about the objective of the study and that it was important to gather information even about those who had not had mastitis. No limit was placed on what length of time had passed since the last child was breastfed. If they preferred not to answer the questionnaire, they were requested to leave it blank and place it in the envelope provided for collection of the questionnaires.

The respondents were asked to give their age, how many children they had given birth to, how many they had breastfed, numbers of times they had mastitis with each child, the age of the baby when mastitis occurred, whether a physician examined them, whether antibiotic treatment was prescribed, whether they continued to breastfeed through the episode(s), what they considered to be the single most important cause of mastitis and what other subsidiary causes they believed affected the development of mastitis. A diagram was included where they were asked to identify the breast (right or left) and the quadrant (upper outside, upper inside, lower outside and lower inside) where the mastitis occurred. They were not asked to give suggestions for care of mastitis since the author of this paper was to talk about mastitis treatment at the meeting, after the questionnaire had been collected.

### Statistical analysis

The material in this study is presented using descriptive statistics; numbers and percentages. For non-normally distributed variables range and median are reported.

## Ethical considerations

The respondents of the questionnaire were not in any state of dependence on the researcher and the president of the Danish Association of Certified Lactation Consultants did not consider there to be any ethical problems for the use of the questionnaire and gave permission for it to be used (personal communication, 27 September, 2011). Since the respondents placed their questionnaires in an envelope, it was possible for those who did not wish to complete the questionnaire to return it blank, without the researcher being aware of this and therefore a completed questionnaire was assumed to have informed consent.

## Results

A total of 91 women attended the meeting and 90 (99%) returned a completed questionnaire. Ages ranged between 33 and 60 years with a median age of 48 years. The total number of babies born to these women was 225 and of these 219 (97%) had ever been breastfed. The range of months of breastfeeding of the last child which the respondents had breastfed was 1–54 months, median 11.5 months.

Thirty women (33%) reported that they had ever experienced mastitis during breastfeeding. The respondents reported episodes of mastitis with 40 of the 219 (18%) breastfed babies born to them. Six women had mastitis with more than one of their babies. A total of 12 women (13%) reported that they had suffered mastitis with their latest breastfed child.

Table 1 shows the ages of the babies when mothers’ mastitis occurred. A total of 40 cases of mastitis were reported and 35 women gave the infant’s age at which mastitis occurred. Of the 35 cases, 94% (*n* =33) occurred before the infant was four months of age and the remaining two cases between four and six months.

**Table 1:** Age of the babies (n = 35) when mothers had mastitis

**Age of baby**	**n (%)**
0-28 days	25 (71)
1-3 months	8 (23)
4-6 months	2 (6)
7-9 months	0
10-12 months	0
>12 months	0

Five women did not specify the baby’s age.

A physician examined 35% (14 of the 40 cases) of the respondents and 93% (*n* = 13) of these were prescribed antibiotics. In 98% (39 of the 40 cases) the respondents reported that they carried on breastfeeding throughout the illness.

**Figure 1:**
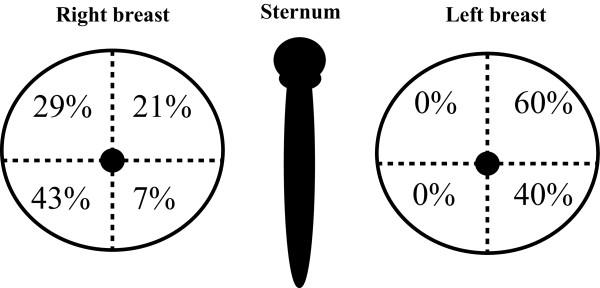
**Representation of the breast segment in which the respondents had experienced mastitis.** Right breast: n = 14. Left breast: n = 10.

Of the 25 who answered the question about which breast was affected, 56% (*n* = 14) had experienced mastitis in their right breast and 44% (*n* = 11) in their left breast (Figure 1). One respondent could not remember which breast segment had been affected. Of the 24 who could remember which segment of the breast had been affected, 83% (*n* = 20) answered that the outside segments (nearest the arms) were affected, upper and lower segments equally. The remaining 17% (*n* = 4) reported affliction of the inside segments.

The respondents were asked to give one factor that they considered to be the most important for the development of mastitis. These are shown in Table 2. Incomplete emptying of the breast caused by too long intervals between feeds or inefficient breastfeeding was by far the most frequently cited factor and the least cited factor was the presence of bacteria.

**Table 2:** The most important predisposing factor for the development of mastitis as reported by the respondents (n = 86)

**Factor**	***n *(%)**
Incomplete emptying of the breast/irregular emptying/inefficient breastfeeding	47 (55)
Attachment problems/wrong position/wrong technique	12 (14)
Cracked nipples	10 (12)
Plugged ducts/blocked milk	5 (6)
Over-production of milk	4 (5)
Being cold	3 (3)
Lack of help/maternal stress	3 (3)
Bacteria	2 (2)

Four respondents did not suggest a predisposing factor.

## Discussion

Surprisingly, the percentage of women in the present study who reported that they had ever had mastitis was exactly the same as the percentage reported in the original Riordan and Nichols study that is, 33% [11]. The greatest problem in interpretation of the results of both the present study and the original study is the fact that no time limit was stated in which the “population at risk” could be identified. The study population cannot be seen as the population at risk for mastitis. Therefore even though in Riordan and Nichols study, 33% of the women stated that they had experienced mastitis during a single lactation period and in the present study 33% said that they had ever had mastitis, these figures cannot be seen as true incidence rates. These were not new cases of mastitis but could have occurred anytime during a period of five years in the older study and during an unlimited period in the new study. We do not know what the population at risk was during a five-year period or how the size of the populations at risk varied over time.

Since it has been demonstrated earlier that none of the symptoms of mastitis correlate with increasing amounts of potential pathogens in the milk [12] and it has been suggested that some women with mastitis may not be febrile [13], the definition of mastitis used in this study allowed for any combination of the common symptoms of mastitis. This could be interpreted as a loose definition. Neither Riordan and Nichol’s study nor the present study required that symptoms should have been experienced for 24 hours or more, which may mean that some of the respondents had episodes of engorgement rather than mastitis. However, the study population in both studies was limited and selective; lactation consultants are likely to be more aware of differences between engorgement and mastitis than other groups of women. The populations in the two studies differed somewhat; the median age of the participants in the present study was 48 years, which was higher than in Riordan and Nichol’s study (median 36 years).

The predisposing factors suggested by the respondents as primarily responsible for symptoms of mastitis are interesting. These women work as breastfeeding consultants and as such have a great deal of clinical experiences on differing breastfeeding problems. Few of the respondents felt that bacteria were the main cause of mastitis despite the fact that the scientific literature often cites *S. aureus* as the main causative organism [14-16]. They answered that irregular and incomplete emptying of the breast was the main cause of mastitis. This would infer that they believe an overfull breast causes high temperature, red, tense and painful breasts. In order for this to be the case, the breast milk would have to overflow or permeate from the milk ducts into the surrounding connective tissue. Researchers have proposed that blocked ducts are responsible for this overflow into the connective tissue [16-18] but a diagnosis of blocked mammary ducts is not easily made. A “chicken or the egg” situation arises if we consider that an inflammatory process in the connective tissue with ensuing swelling might well exert pressure on the milk ducts making drainage difficult. Whether blocked milk ducts are a cause of or a product of lactational mastitis has yet to be scientifically confirmed.

It was notable that 33% reported being treated with antibiotics. This figure is relatively low in comparison to other international studies, which show that 77% - 97% of women are prescribed antibiotics [2,9,19]. Of the women in this study who were put in contact with a physician a great majority of them were prescribed antibiotics. The present study was carried out in Denmark and it has been shown that in neighbouring Sweden approximately 15% are prescribed antibiotics [12]. These findings might represent a cultural tendency to lessen the use of antibiotics for this group of patients since they are often managed by midwives and lactation consultants who are used to the sight of very red and inflamed breasts. Their experience may have taught them that although the women feel very ill, spontaneous recovery is common.

In an audit of mastitis cases from Australia, it was shown that mastitis occurred most often unilaterally and equally in both left and right breasts [13]. Similarly, results from the present study show a relatively equal distribution between right and left sides. The results shown in Figure 1 indicate that in a large proportion of women, inflammation occurs in the outside quadrants of the breasts.

An American review of the scientific literature has reported a wide range of incidences from 2% to 33% [20] and the 33% incidence reported in the review is also included in a World Health Organisation report on mastitis [21]. Science is dynamic; methods used and conclusions drawn change over time. Retrospective studies have during recent years become less interesting from a scientific view point due to the unsure nature of the results caused by re-call bias. It is therefore relevant to challenge the reported 33% incidence of mastitis. I propose that the term incidence was incorrectly used in Riordan and Nichols paper and for that reason the incidence rate of 33% should not continue to be cited by other researchers.

## Conclusions

The population of breastfeeding women is not easily measured because of ever-fluctuating initiation levels and levels of breastfeeding cessation in the communities where breastfeeding is measured. Therefore, by the rule that says that we must be able to identify the population at risk, the incidence of mastitis can only ever be an approximation. Despite this, researchers must strive to be as exact as possible when reporting incidences and should attempt to evaluate the true population at risk. Well-designed studies in many different global locations are needed before any conclusions can been drawn about the range of incidences of mastitis.

## Competing interests

This study was not funded. The author declares that there are no existing conflicts of interest.
